# FuseAD: Unsupervised Anomaly Detection in Streaming Sensors Data by Fusing Statistical and Deep Learning Models

**DOI:** 10.3390/s19112451

**Published:** 2019-05-29

**Authors:** Mohsin Munir, Shoaib Ahmed Siddiqui, Muhammad Ali Chattha, Andreas Dengel, Sheraz Ahmed

**Affiliations:** 1 German Research Center for Artificial Intelligence (DFKI) GmbH, 67663 Kaiserslautern, Germany; shoaib_ahmed.siddiqui@dfki.de (S.A.S.); muhammad_ali.chattha@dfki.de (M.A.C.); andreas.dengel@dfki.de (A.D.); sheraz.ahmed@dfki.de (S.A.); 2 Fachbereich Informatik, Technische Universität Kaiserslautern, 67663 Kaiserslautern, Germany; 3 School of Electrical Engineering and Computer Science (SEECS), National University of Sciences and Technology (NUST), 44000 Islamabad, Pakistan

**Keywords:** time-series analysis, anomaly detection, deep neural networks, statistical models, model fusion, sensor data

## Abstract

The need for robust unsupervised anomaly detection in streaming data is increasing rapidly in the current era of smart devices, where enormous data are gathered from numerous sensors. These sensors record the internal state of a machine, the external environment, and the interaction of machines with other machines and humans. It is of prime importance to leverage this information in order to minimize downtime of machines, or even avoid downtime completely by constant monitoring. Since each device generates a different type of streaming data, it is normally the case that a specific kind of anomaly detection technique performs better than the others depending on the data type. For some types of data and use-cases, statistical anomaly detection techniques work better, whereas for others, deep learning-based techniques are preferred. In this paper, we present a novel anomaly detection technique, FuseAD, which takes advantage of both statistical and deep-learning-based approaches by fusing them together in a residual fashion. The obtained results show an increase in area under the curve (AUC) as compared to state-of-the-art anomaly detection methods when FuseAD is tested on a publicly available dataset (Yahoo Webscope benchmark). The obtained results advocate that this fusion-based technique can obtain the best of both worlds by combining their strengths and complementing their weaknesses. We also perform an ablation study to quantify the contribution of the individual components in FuseAD, i.e., the statistical ARIMA model as well as the deep-learning-based convolutional neural network (CNN) model.

## 1. Introduction

In the current era of smart and connected devices, there are more than 12 billion IoT devices, and it is estimated that there will be over 20–25 billion “things” as part of the IoT environment by 2025 [1,2]. The sensors in IoT devices are continuously generating streaming data that can be analyzed to (a) monitor device health; (b) foresee the problems which could arise in the device; and (c) make the device intelligent by adapting to varying behaviors. Nowadays, a common use of the streaming data is to detect the anomalies in a system for fault diagnosis and predictive analytics [3,4,5,6]. The connected devices are generating a large amount of data per second, so it is nearly impossible to analyze them manually. Therefore, it is vital to have a robust anomaly detection technique for streaming data.

An anomaly is an outlier, which Hawkins [7] defined as an observation that deviates so significantly from other observations as to arouse suspicion that it was generated by a different mechanism. The term “anomaly detection” is context-dependent, and its meaning varies from domain to domain. For example, an unauthorized interference in a network is an anomaly, whereas a car is considered as an anomaly inside a park. Anomaly detection is also referred to as intrusion detection, fault detection, fraud detection, and outlier detection. Considering the importance of anomaly detection and its wide area of applicability, there exist many methods for anomaly detection in general [8,9,10,11] and for streaming data in particular [4,12,13,14]. In the context of streaming data, some methods have shown their supremacy over other methods for a particular set of use-cases. However, no such method exists that can be deployed in every use-case [15]. Statistical models have proved to be quite effective in some areas for anomaly detection, while deep-learning-based anomaly detection techniques have shown promising results in other domains. Each technique has its own advantages and limitations. Nowadays, much of the research is focused on deep-learning-based approaches, whereas statistical models are widely accepted in a practical environment, i.e., in industry, specifically due to their transparency. Both techniques are well suited for anomaly detection, but the choice of a technique depends on the use-case and the type of data. To fill this gap of picking “one” model for a specific use-case and to increase the accuracy of the detected anomalies, we propose a fusion technique called FuseAD that is based on the idea of fusing statistical and deep learning models for anomaly detection. By combining these two disjoint worlds, we can profit from both. The main advantage of such a fusion is that where one model is weak, the strength of the other model plays its role and improves the overall process of anomaly detection. In particular, the contributions of this paper are as follows:

- A novel fusion method for deep-learning-based and statistical-model-based anomaly detection techniques. In contrast to the ensembling-based anomaly detection methods in which one out of different forecasting results is picked based on the lowest error, the proposed residual scheme lets the network learn how to produce the best forecasting outcome based on two different kinds of models. In addition, the fusion mechanism enables the network to complement the strengths of the underlying two disjoint models by fusing the information encapsulated in them. As a result, the fused network performs better in cases where a single model is unable to produce good results.
- Extensive evaluation of different distance-based, machine-learning-based, and deep-learning-based anomaly detection methods including iForest [9], one-class support vector machine (OCSVM) [16], local outlier factor (LOF) [8], principal compnent analysis (PCA) [17], TwitterAD [12], DeepAnT [13], Bayes ChangePT [18], Context OSE [19], EXPoSE [20], HTM Java [19], NUMENTA [14], Relative Entropy [21], Skyline [19], Twitter ADVec [12], and Windowed Gaussian [19] on two anomaly detection benchmarks. These benchmarks contain a total of 425 time-series.
- An ablation study in order to identify the contribution of the different components in FuseAD. In this study, we highlight the significance of using the fused model by comparing the results with each individual model.

The rest of the paper is structured as follows. We first provide a glimpse of the previous work in the direction of traditional and deep-learning-based anomaly detection in Section 2. We then provide details regarding the proposed method (FuseAD) in Section 3. We define the experimental protocol in Section 4. The obtained results are compared and discussed in Section 5. In Section 6, we present the results from the ablation study that is performed on FuseAD. Finally, we conclude the paper with future perspectives in Section 7.

## 2. Literature Review

There exist a lot of anomaly detection techniques for detecting anomalies in images, videos, and sensor data [15]. In this section, we focus on the anomaly detection techniques that are commonly applied to sensor data. Generally, the anomaly detection techniques are categorized based on the following criteria [15,22,23,24]:

- **Type of anomaly**: point anomaly, contextual anomaly, and collective anomaly.
- **Availability of labels**: supervised, unsupervised, and semi-supervised.
- **Type of employed model**: linear models, statistical models, probabilistic models, clustering-based, nearest-neighbors-based, density-based, and deep-learning-based, etc.
- **Applications**: fraud detection, surveillance, industrial damage detection, medical anomaly detection, and intrusion detection, etc.

We categorize the anomaly detection methods into traditional distance-based and deep-learning-based anomaly detection techniques. *k*-nearest neighbor (*k*-NN) is a distance-based unsupervised anomaly detection technique proposed by Ramaswamy et al. (2000) [25]. This technique is based on the distance of a point from its *k*th nearest neighbor. Each point is ranked on the basis of its distance to the *k*th nearest neighbor, and the top *n* points in this ranking are declared as outliers. This technique is highly dependent on the value of *k* and may fail when there are too few neighbors around normal data points. A widely used density-based local outlier detection method, local outlier factor (LOF), was proposed by Breunig et al. (2000) [8]. The outlier factor indicates how isolated an object is from its surrounding neighbors based on a concept of local density. The locality is computed based on the distance to *k* nearest neighbors. By comparing the local density of an object to its neighbors, an anomalous object is detected. The anomalous points have lower density as compared to their neighbors. However, LOF faces some issues in scenarios where normal data points are distributed in a linearly connected way, in contrast to the assumed distribution in a spherical manner. The improved variants and extensions of LOF are connectivity-based outlier factor (COF) and influenced outlierness (INFLO), which are proposed by Tang et al. (2002) [26] and Jin et al. (2006) [27], respectively. To identify diverse attacks in an internet network, Vasiliadis et al. (2011) [28] introduced an architecture for network intrusion detection systems. Generally, for streaming data, the anomaly detection methods consist of two modules, a value at the next timestamp is forecasted first, and then it is compared with the actual value to mark the data point as normal or anomalous [13,29]. In most of such cases [30,31], the forecasting module is based on auto-regressive integrated moving average (ARIMA), which is a generalization model of auto-regressive moving average (ARMA) [32]. It consists of three components: (i) the auto-regression part uses the dependent relation between an observation and prior (lagged) values; (ii) the moving average part incorporates the dependency between an observation and a residual error from a model applied to lagged observations; and (iii) the integrated part represents the difference between the observed values and the previous values.

With the rapid increase in the applicability of artificial neural networks (ANN) in different domains like automotive [33], government [34], health [35], security & surveillance [36], more deep-learning-based anomaly detection techniques are being introduced. Malhotra et al. (2015) [37] introduced long short-term memory (LSTM)-based anomaly detection technique for time-series data. They train stacked LSTM on non-anomalous data and use it as a predictor over different time-stamps. The prediction errors are further modeled to obtain the likelihood of anomalous sequences. Chauhan and Vig (2015) [38] also proposed a similar approach based on deep LSTMs. The anomalous pattern detection technique for multivariate clinical time-series proposed by Lipton et al. (2016) [39] is also based on LSTMs. They have shown in their study that an LSTM trained on raw data is superior to an MLP trained on hand-engineered features. Zheng et al. (2014) [40] proposed a CNN-based approach for multivariate time-series classification problems. Each channel of the proposed multi-channel deep CNN learns features individually when multivariate data is presented and classifies it as a normal or anomalous sequence. Munir et al. (2019) [13] also proposed a CNN-based anomaly detection technique for time-series data, known as DeepAnT. In the area of network monitoring, Lopez-Martin et al. (2017) [41] introduced a method for network traffic classification. They combined LSTM and CNN models to better classify the network sequences without providing any hand-engineered features. In order to apply CNN to time-series data, they proposed an approach to render the data as an associated pseudo-image. In contrast to all of the aforementioned ANN-based anomaly detection techniques, DeepAnT detects point and contextual anomalies. It is relatively difficult to precisely detect point anomalies in streaming data, as compared to the traditional classification of a sequence into normal or abnormal classes, because of the presence of seasonality and trend.

Du et al. (2017) [42] introduced a method for network fusion in which they fuse together the soft metrics from different networks to generate the final confidence score. However, their approach is not directly applicable in our case, where we aim to fuse a statistical model and a deep learning model to get benefits from the two different approaches. The anomaly detection technique proposed by Buda et al. (2018) [29] merges the predictions from different LSTM models and statistical models in the forecasting module. They proposed two approaches for merging the results of time series forecasting. In the single-step merge approach, each model forecasts the next value and the forecasted value with the lowest root mean square error (RMSE) is selected. In the vote merge approach, the best forecasting model is voted on based on the training data. In their approach, each model works independently, and the best forecast is selected from a number of models. However, this is not the case in the technique that we propose in this paper. In FuseAD, a network learns itself how and when to fuse the statistical and deep learning forecasting to generate the best forecasting results.

## 3. Methodology

In this section, we explain the two forecasting models from the statistical and deep learning domains. ARIMA and CNN forecasting models are building blocks of the proposed FuseAD and are combined in a way to get benefit from each other. These two models are used to forecast the next time-stamp in a given time-series. The forecasted value is further passed to an anomaly detector module that marks a data point as normal or anomalous.

### 3.1. Statistical Model (ARIMA)

ARIMA is a well-known and widely used statistical technique for time-series forecasting [43]. We used ARIMA as our statistical model since it has been employed successfully for a wide range of use-cases in the industry to handle time-series regression tasks [44,45,46]. To get the best out of the ARIMA model, it is important to find the right set of parameters for a given time-series. Non-seasonal ARIMA models are denoted as *ARIMA(p, d, q)*, where *p*, *d*, and *q* are non-negative integer parameters. Lag order (p) is the number of lag observations included in the model, the degree of differencing (d) is the number of nonseasonal differences needed to make the series stationary, and the moving average window size (q) is the number of lagged forecast errors in the prediction.

### 3.2. Deep-Learning-Based Model (CNN)

CNN has proved its superiority over other ANN variants in many computer vision applications [47] and also in time-series anomaly detection applications [13]. The CNN model is composed of 2 convolutional layers, where the first convolutional layer is followed by a max-pooling layer. Finally, the output is generated through a fully-connected layer producing continuous valued outputs. The network is trained through mean absolute error (MAE) as the loss function since the output is real-valued. We keep the architecture simple with a minimal number of parameters in order to make sure that the network can be successfully constrained to a reasonable solution with a very limited amount of data, which is a common case in publicly available time-series datasets. CNN is commonly used as a directly forecasting model. This formulation can be represented as:

(1) $${\hat{x}}_{t}=\Phi \left(\left[x_{t-w},\ldots ,x_{t-1}\right]\right),$$

where $\Phi (\left[x_{t-w},\ldots ,x_{t-1}\right])$ indicates the output of the network and ${\hat{x}}_{t}$ indicates the output of the system, which are same in this case. In Equation (1), *w* is the size of the history window. Therefore, the network learns a mapping from the input space X to the output space Y.

### 3.3. FuseAD: The Proposed Method

**Figure 1:**
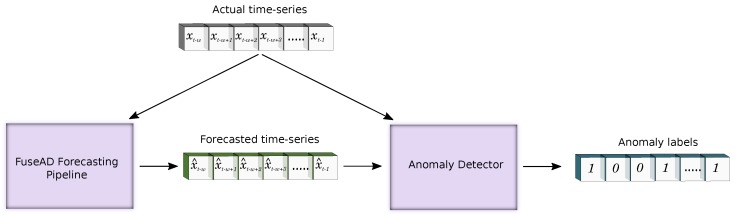
FuseAD overview: The system consists of two modules, the forecasting pipeline and an anomaly detector.

The proposed technique consists of two modules as shown in Figure 1. The first module is called the forecasting pipeline. An actual time-series is fed into this module and it generates a forecasted time-series. This forecasted time-series is further passed to an anomaly detector module, which is responsible for detecting anomalies. Based on the forecasted time-series and the actual time-series, the anomaly Detector marks each time-stamp as normal or abnormal. Both modules are discussed in detail in this section.

#### 3.3.1. Forecasting Pipeline

Instead of using both statistical and deep-learning-based models in isolation, we combine these models in a novel residual learning scheme. This enables the system to complement each model’s strengths by using the information encapsulated in the other system. In this formulation, instead of treating the CNN as a mapping from the input space X to the output space Y, we consider it to be a mapping from the input space X to an intermediate space Z∈R. We then add an offset (the output of ARIMA) to transform it back to the output space Y.

**Figure 2:**
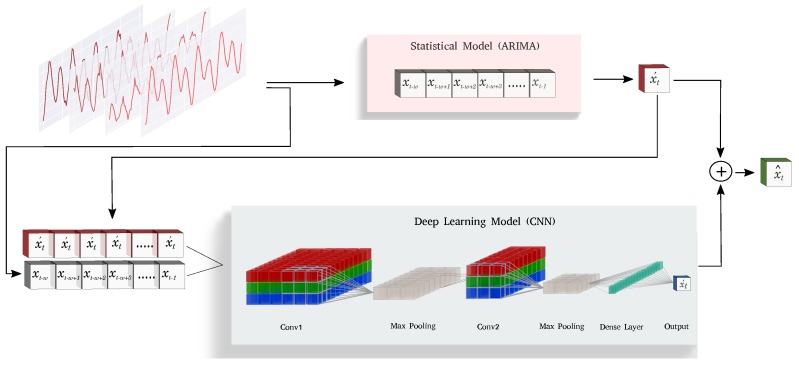
FuseAD forecasting pipeline.

The new formulation, therefore, is a mixture of the two models in a residual scheme. We simply augment the output of the CNN by introducing a summation layer in the end. In this way, the output of CNN can be considered as a correction term for the output of the ARIMA model. In a case where the output of ARIMA is accurate, CNN can suppress its output in order to retain the prediction made by ARIMA. On the other hand, when the prediction is significantly off, the network can generate large offsets in order to compensate for the error made by the ARIMA model. In this way, the network itself can decide its reliance on the output of ARIMA during training to adapt its behavior so as to overcome its limitations. The new formulation can be written as:

(2) $${\hat{x}}_{t}=\Phi \left(\left[x_{t-w},\ldots ,x_{t-1};x_{t}^{\prime }\right]\right)+x_{t}^{\prime },$$

where $x_{t}^{\prime }$ indicates the output from ARIMA, $\Phi (\left[x_{t-w},\ldots ,x_{t-1};x_{t}^{\prime }\right])$ indicates the output of the CNN network, and ${\hat{x}}_{t}$ indicates the output of the whole forecasting pipeline. It is important to note that we condition the output of the CNN on the output of ARIMA. This step is essential as we want to generate an offset or a correction term to the prediction made by ARIMA; therefore, the network should have access to the prediction made by ARIMA. The proposed forecasting pipeline is shown in Figure 2.

There can be many possible strategies to achieve this conditioning. We resorted to the easiest possible formulation by directly stacking the prediction of ARIMA in a different channel to the actual signal. This enables the network to keep in consideration the conditioning term at every point in the sequence. This conditioning strategy might be problematic for cases where multi-step-ahead prediction is desired; however, we restrict ourselves to single-step-ahead forecasting, which is required for anomaly detection scenarios. This is an unsupervised learning technique, which can benefit from a large amount of unlabeled data. Instead of ARIMA and CNN, other statistical and deep-learning-based forecasting models can also be used to make predictions.

#### 3.3.2. Anomaly Detector

When the forecasting model generates a prediction, it is passed to the anomaly detection module. Based on the anomaly score produced by this module, a time-stamp is marked as a normal (0) or an abnormal (1) instance. The anomaly score (shown in the lower plot of Figure 4) is computed based on the distance between the predicted value and the actual value. We use Euclidean distance (as mentioned in [13]) given in Equation (3) as an anomaly score.

(3) $$\left(x_{t},{\hat{x}}_{t}\right)=\sqrt{{\left(x_{t}-{\hat{x}}_{t}\right)}^{2}}$$

where $x_{t}$ is the actual value and ${\hat{x}}_{t}$ is the predicted value by the system computed using Equation (2).

## 4. Experimental Setups

We have evaluated the proposed technique on two anomaly detection benchmarks that consist of real and synthetic streaming data. Based on these benchmarks, we compared the state-of-the-art anomaly detection methods with FuseAD. In this section, we provide details of the used benchmarks, the settings in which they are used, and the evaluation metric we used for the comparative analysis.

### 4.1. Yahoo Webscope Dataset

#### 4.1.1. Dataset Description

Yahoo Webscope [4] is an open-source time-series anomaly detection benchmark. This benchmark is further divided into four sub-benchmarks: A1, A2, A3, and A4, which consist of real and synthetic data. There are 367 time-series in this dataset, where each sequence is comprised of 1420–1680 instances. The real dataset (A1) contains Yahoo membership login data, which tracks the aggregate status of user logins to their network. The synthetic time-series (A2–A4) are generated by specifying the length, a number of anomalies, noise level, trend, anomaly type, and seasonality. Anomaly labels are editorially or synthetically generated by the publisher and are provided with the dataset.

#### 4.1.2. Experimental Setting

We have used *Auto ARIMA* [43] to get the best ARIMA model for forecasting as ARIMA requires data-specific tuning to obtain the best results. Since each time-series has a different trend, change point, and seasonality, we tune the ARIMA model separately for each time-series. The ARIMA models with different sets of parameters are tuned on 40% of a time-series, and the best model is selected based on the lowest Akaike information criterion (AIC) value. The best model is used to make a single-step-ahead (horizon of 1) forecast on the rest of the 60% of the data. For CNN-based forecasting, we have used the same hyper-parameters as mentioned in [13]. The same 40/60 data split as used for the ARIMA model is used here. We have used five other anomaly detection methods for comparison with FuseAD on the Yahoo dataset. For all these methods, we used 40% of the data for training and the remaining 60% for testing.

### 4.2. NAB Dataset

#### 4.2.1. Dataset Description

**Figure 3:**
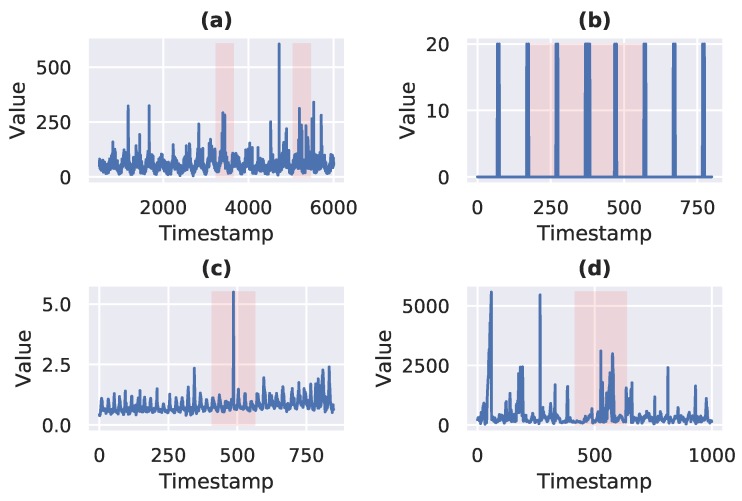
(**a**) Real Tweets (Twitter_volume_AMZN), (**b**) Artificial With Anomaly (art _increase_spike_density); (**c**) Real Ad Exchange (exchange-3_cpm_results); (**d**) Real Traffic (TravelTime_451_whole). Snippets of Numenta Anomaly Benchmark (NAB) time-series from different domains are plotted. Actual time-series are shown in blue, whereas the highlighted area shows an anomaly window.

The Numenta Anomaly Benchmark (NAB) [14] is an open-source streaming anomaly detection benchmark introduced by Numenta. This dataset contains streaming data from the following domains: internet traffic, advertisement, cloud service, and automotive traffic. There are 58 time-series in this dataset, where each sequence is comprised of 1000–22,000 instances. This benchmark contains both real and artificial time-series. A window of defined size (10% of the sequence length) is labeled as an anomalous window if anomalous points are present in that window. In most of the cases, the actual anomalous data points in an anomalous window are 2–3, but the whole window is marked as anomalous. The snippets of a few time-series given in the NAB dataset are shown in Figure 3. The actual time-series is shown in blue, whereas the highlighted region shows anomaly labels. The benchmark is labeled either based on the known root cause of an anomaly or by following the defined anomaly labeling procedure by the publisher. We used the following short forms of the domain names according to which time-series are categorized in this dataset: Artificial No Anomaly –> Artificial-nA, Artificial With Anomaly –> Artificial-wA, Real Ad Exchange –> Real-AdE, Real AWS Cloud Watch –> Real-AWS, Real Known Cause –> Real-KC, Real Traffic –> Real-Tr, Real Tweets –> Real-Tw. These short forms are used in Tables 2 and 4.

#### 4.2.2. Experimental Setting

We have used 40% of each time-series of the NAB dataset to train FuseAD in the same fashion as is done for the Yahoo dataset. We compared FuseAD with nine other anomaly detection methods for the evaluation. The results of all the algorithms are reported on 60% of the actual time-series (test data).

### 4.3. Evaluation Metric

We have used the receiver operating characteristic (ROC) curve for comparing the proposed method with the state-of-the-art anomaly detection methods. This metric helps in uncovering the maximum potential of an algorithm whose performance is dependent on selecting the best threshold. The best threshold depends upon the needs and other criteria for a use-case such as the maximum number of true positives and the minimum number of false alarms. The ROC curve is created by plotting the true positive rate (TPR) vs. the false positive rate (FPR) for different threshold settings, providing a broader overview of an algorithm’s classification capability. We have used area under the ROC curve (AUC) to provide an aggregated measure of the used models’ performance. AUC values near 1 represent a good measure of separability. Average AUC per domain in the NAB dataset and per sub-benchmark in the Yahoo dataset are reported as both datasets contain multiple time-series.

## 5. Results

**Figure 4:**
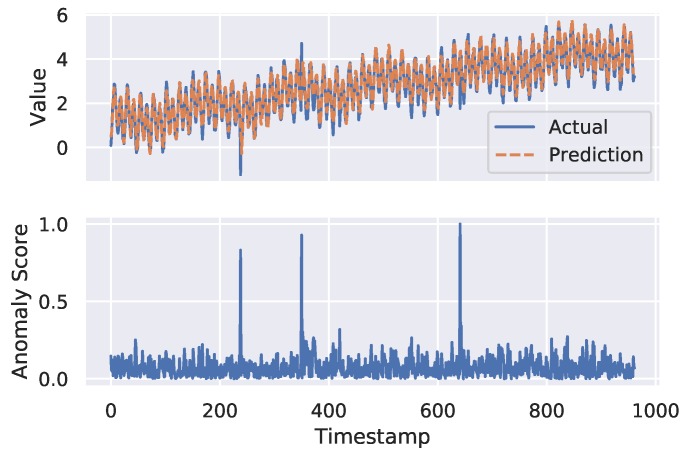
Forecasting and anomaly detection results of FuseAD on the TS11 time-series from the Yahoo A3 sub-benchmark. The upper plot shows the actual time-series and forecasting results on test data, whereas the lower plot shows the anomaly score at each time-stamp. The anomaly label (i.e., 1) is assigned to data points that have a high anomaly score.

**Figure 5:**
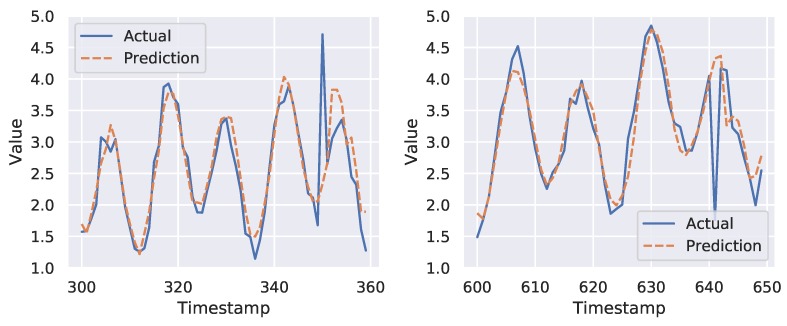
Zoomed-in plots of two out of three anomalies detected in Figure 4. It shows that FuseAD is capable of correctly detecting point anomalies in streaming data where traditional anomaly detection methods fail normally.

Figure 4 shows the forecasting and anomaly detection results of FuseAD on a sample time-series from the Yahoo dataset. In the upper plot of this figure, the actual time-series is shown in blue, whereas the predictions are shown in orange. It can be seen in this plot that the network is able to learn the time-series trend and cycles. In the lower plot of this figure, the anomaly score per time-stamp is given. FuseAD detected three instances with a high anomaly score in the mentioned time-series. The zoomed-in plots of two of the detected anomalies are shown in Figure 5. In these plots, the normal behavior learned by the model is predicted per time-stamp, which deviates from the observed behavior at index 350 and 641, respectively. It is clear from these zoomed-in plots that FuseAD is able to precisely detect point anomalies that are otherwise easily overlooked by traditional distance-based and density-based anomaly detection methods in time-series data. We compared the AUC of FuseAD with other state-of-the-art anomaly detection methods including LOF [8], iForest [9], OCSVM [16], PCA [17], Twitter anomaly detection (TwitterAD) [12], and DeepAnT [13] on Yahoo Webscope dataset. Table 1 compares the average AUCs of FuseAD with the mentioned methods on the Yahoo Webscope dataset. It can be seen in this table that FuseAD has outperformed the other methods.

Table 2 shows comparative results of FuseAD and other streaming and kernel-based anomaly detection methods on the NAB dataset. The average AUC per domain is reported for each anomaly detection method. We used DeepAnT [13], Bayes ChangePT [18], Context OSE [19], EXPoSE [20], HTM Java [19], NUMENTA [14], Relative Entropy [21], Skyline [19], Twitter ADVec [12], and Windowed Gaussian [19] anomaly detection methods for the comparison. It can be observed in this table that there is no single anomaly detection method that outperforms others. There is high variance in the performance of every method on different datasets, where the average performance of every method is close to random. It is not because none of these methods are capable of detecting anomalies in streaming data, but it is mainly due to the poor labeling mechanism used in the NAB dataset. It can be seen in Figure 3b,c that there is a small number of data points that are actually anomalous, but the NAB labeling mechanism has labeled all the data points in an anomaly window as anomalous data points. Most of the data points in these windows are apparently normal. On the other hand, there are no anomalous data points in the anomaly windows shown in Figure 3a,d, whereas the actual anomalous data points are not labeled as anomalous. Due to these issues and other issues mentioned by Singh and Olinsky (2017) [48], it is hard for an anomaly detection method to have a high AUC under these circumstances. Thus, most of the methods end up conducting random detection of anomalies in the NAB dataset.

## 6. Ablation Study

We performed an ablation study on the FuseAD framework in order to identify the contribution of different components in the overall pipeline. FuseAD combines the ARIMA statistical model and the CNN deep learning model in a novel residual scheme. This combined prediction is fed to the anomaly detection module, which decides if an instance is normal or abnormal. In the ablation study, we remove one of the forecasting modules, i.e., either the CNN or the ARIMA model, to check the influence of that model on the overall anomaly detection process.

We first remove the CNN from the formulation presented in Equation (2). This leaves the final prediction to only rely on the prediction made by the forecasting model. Therefore, the new formulation becomes:

(4) $${\hat{x}}_{t}=x_{t}^{{}^{\prime }},$$

where $x_{t}^{{}^{\prime }}$ represents the prediction made by the statistical model and ${\hat{x}}_{t}$ represents the overall output of the system. Similarly, we remove the ARIMA model from the original formulation (Equation 2). In this case, CNN learns the complete input-to-output space projection. This formulation can be written as:

(5) $${\hat{x}}_{t}=\Phi \left(\left[x_{t-w},\ldots ,x_{t-1}\right]\right),$$

where $\Phi (\left[x_{t-w},\ldots ,x_{t-1}\right])$ represents the prediction made by the network and ${\hat{x}}_{t}$ represents the overall output of the system. We also remove the conditioning term since there is no statistical model available in this case. This formulation is exactly the same as the one from Equation (1) where we trained a CNN for forecasting, except for the presence of the FuseAD anomaly detection module in the end.

We use the same data splits as used by FuseAD for training the respective models, in order for the comparison to be fair. The results from the ablation study on the Yahoo Webscope dataset are presented in Table 3. It is apparent from the table that a novel combination of the components leveraged by FuseAD significantly improves performance in most of the cases. The results from the ablation for the NAB dataset are presented in Table 4. As is evident from the table, the results are again chaotic due to the poor quality of the dataset itself.

**Figure 6:**
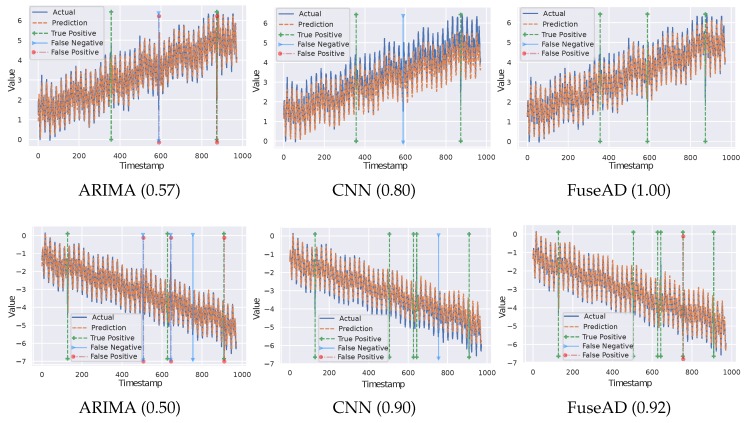
Comparative analysis of FuseAD and anomalies detected by ARIMA and CNN models on two sample time-series. The first row shows results on TS29 and the second row shows results on TS18 from the A3 Yahoo benchmark. Respective F-scores are shown in brackets.

To highlight the importance of fusing statistical and deep learning models, comparative analyses of FuseAD based on the ablation study are shown in Figure 6 on two different time-series from the Yahoo dataset. First, we remove the CNN forecasting model from FuseAD and show anomaly detection results generated by the ARIMA model (first column). Then, we remove the ARIMA forecasting model, which means only the CNN model is used for forecasting in FuseAD (second column). Finally, we use both forecasting models in actual FuseAD setting (third column). We use the same parameters as mentioned in [13] to detect anomalies and calculate an F-score (mentioned in brackets in Figure 6). It can be observed in these plots that in both ARIMA plots, all of the anomalies are not detected (false negatives) and there are many false alarms (false positives) too. In ARIMA cases, the false alarms are given just after the actual anomalous data point (normally 1–2 indexes), which shows that the forecasting is not very robust when there exist cycles and trends. In CNN plots, there are no false alarms, but there are false negatives. FuseAD results presented in this figure show that an anomaly that is not detected by using a model in isolation is detected by fusing both models. By learning from both models, the number of false alarms is also reduced in FuseAD.

## 7. Conclusions

For detecting anomalies in streaming data generated by IoT-based sensors, traditional statistical models are widely used, specifically due to their transparency. However, anomaly detection based on deep learning models has shown significant improvement in terms of performance. Both types of models have some restrictions, and no method scales perfectly to every use-case. To take advantage of both types of models, we proposed FuseAD, a method to fuse statistical and deep-learning-based models for time-series anomaly detection. In contrast to other model fusion methods, we fused two disjointed models in a novel residual learning scheme where the network learns itself when to give preference to a particular model’s forecast. This method enables the system to gain the strengths of both models. It is evident from the obtained results that FuseAD outperforms state-of-the-art anomaly detection methods on the Yahoo Webscope dataset. The ablation study and comparative analysis show that FuseAD indeed improved performance in comparison to the use of isolated components (statistical or deep learning model). In the future, we aim to extend the evaluation of this method to multi-variant time-series datasets by fusing other traditional distance-based anomaly detection methods (e.g., LOF and k-NN) and other deep learning models (e.g., LSTM).

## Figures and Tables

**Table 1:** Comparative evaluation of state-of-the-art anomaly detection methods on the Yahoo Webscope dataset. Average AUC per sub-benchmark is shown in this table.

Benchmark	iForest [9]	OCSVM [16]	LOF [8]	PCA [17]	TwitterAD [12]	DeepAnT [13]	FuseAD
A1	0.8888	0.8159	0.9037	0.8363	0.8239	0.8976	**0.9471**
A2	0.6620	0.6172	0.9011	0.9234	0.5000	0.9614	**0.9993**
A3	0.6279	0.5972	0.6405	0.6278	0.6176	0.9283	**0.9987**
A4	0.6327	0.6036	0.6403	0.6100	0.6534	0.8597	**0.9657**

**Table 2:** Comparative evaluation of anomaly detection methods on the NAB dataset. Average AUC per domain is reported here. Bold numbers show highest AUC in a particular domain.

	Bayes ChangePT [18]	Context OSE [19]	EXPoSE [20]	HTM Java [19]	NUMENTA [14]	Relative Entropy [21]	Skyline [19]	Twitter ADVec [12]	Windowed Gaussian [19]	DeepAnt [13]	FuseAD
Artificial-nA	0	0	0	0	0	0	0	0	0	0	0
Artificial-wA	0.502	0.316	0.5144	**0.653**	0.531	0.505	0.558	0.503	0.406	0.555	0.544
Real-AdE	0.509	0.307	0.581	0.568	0.576	0.505	0.534	0.504	0.538	0.562	**0.588**
Real-AWS	0.499	0.311	0.594	0.587	0.542	0.506	0.602	0.503	**0.614**	0.583	0.572
Real-KC	0.501	0.486	0.533	0.584	0.590	0.503	**0.610**	0.504	0.572	0.601	0.587
Real-Tr	0.507	0.310	0.613	**0.691**	0.679	0.508	0.556	0.505	0.553	0.637	0.619
Real-Tw	0.498	0.304	**0.594**	0.549	0.586	0.500	0.559	0.505	0.560	0.554	0.546

**Table 3:** Ablation study on the Yahoo Webscope dataset.

	A1			A2			A3			A4		
ARIMA	✓		✓	✓		✓	✓		✓	✓		✓
CNN		✓	✓		✓	✓		✓	✓		✓	✓
AUC	0.920	0.936	**0.947**	**0.999**	**0.999**	**0.999**	0.992	0.986	**0.998**	0.949	0.928	**0.965**

**Table 4:** Ablation study on the NAB dataset.

	Artificial-nA			Artificial-wA			Read-AdE			Real-AWS			Real-KC			Real-Tr			Real-Tw		
ARIMA	✓		✓	✓		✓	✓		✓	✓		✓	✓		✓	✓		✓	✓		✓
CNN		✓	✓		✓	✓		✓	✓		✓	✓		✓	✓		✓	✓		✓	✓
AUC	0	0	0	0.49	0.53	**0.54**	0.56	**0.58**	**0.58**	0.55	**0.58**	0.57	0.50	**0.60**	0.58	0.58	**0.61**	**0.61**	**0.55**	**0.55**	0.54

## Abbreviations

The following abbreviations are used in this manuscript:

AUC	Area under curve
NAB	Numenta Anomaly Benchmark
ROC	Receiver operating characteristic curve
LOF	Local outlier factor
COF	Connectivity-based outlier factor
INFLO	Influenced outlierness
TPR	True positive rate
FPR	False positive rate
AIC	Akaike information criterion
ARIMA	Auto-regressive integrated moving average
ANN	Artificial neural network
CNN	Convolutional neural network
ARMA	Auto-regressive moving average
RMSE	Root mean square error
MAE	Mean absolute error
OCSVM	One-class support vector machine
PCA	Principle component analysis
LSTM	Long short-term memory
